# Effect of various mulches on soil physico—Chemical properties and tree growth (*Sophora japonica*) in urban tree pits

**DOI:** 10.1371/journal.pone.0210777

**Published:** 2019-02-06

**Authors:** Bingpeng Qu, Yuanxin Liu, Xiangyang Sun, Suyan Li, Xinyu Wang, Kaiyi Xiong, Binghui Yun, Hua Zhang

**Affiliations:** 1 Forestry College, Beijing Forestry University, Beijing, China; 2 Tianjin LVYIN Landscape and Ecology Construction Co., Ltd, Tianjin, China; Assam University, INDIA

## Abstract

Mulching is a widely employed soil management practice. The mulches used have variable effects on the soil properties and plant growth. In China, mulches are used to cover bare soil at a few places in landscapes, where most of the soil is uncovered, especially in tree pits. As to improve our understanding on the effect of various mulches on soil properties and tree growth after two years of the treatment justifying its implication in soil fertility and tree growth. A comparison study was conducted to determine the effects of inorganic (cobblestone—CB; water permeable brick—WPB), organic (pine bark—PB; green waste compost—GWC), and living (turf grass—TG) mulches on soil physical and chemical properties at three different depths (0–10 cm, 10–20 cm, and 20–40cm), and on tree growth (*Sophora japonica*) in urban tree pits. Soil moisture was measured once a month in 2015.The soil samples were collected from the tree pits two years after mulching and used to evaluate the physical and chemical properties. Further, trunk diameter and tree height were determined once a year. During the most months, all types of mulches significantly affected the moisture content of the soil at all the depths analyzed. In July and August, however, the moisture content of PB and TG treated soil decreased when compared with that of unmulched bare soil. Two years after mulching, the bulk density of the soil treated with PB, GWC, and TG was significantly affected at10–20 cm, with GWC exhibiting a relatively better effect. The treatments with PB, GWC, and TG also improved the total porosity, macroporosity, and microporosity of the soil at lower depths. Further, WPB worsened the bulk density and porosity of the soil, elevating the pH at lower depths. The organic matter, total N, mineral N, available P, and available K contents of the soil at lower depths increased when mulched with organic material. Turf grass significantly increased only the total N and available K at 0–10 and 10–20 cm. There was no significant difference in the soil properties among the treatments at 20–40 cm. Furthermore, the trunk diameter and tree height were not affected by the mulches two years after mulching. In conclusion, organic mulches especially GWC, not only increased soil fertility significantly but improved soil physical characters (0–10 cm depth) comparing to other mulches, are suitable to cover bare soil in urban tree pits.

## Introduction

Mulches have been widely used in agriculture lands, orchards, forests, and landscapes in many parts of the world [1]. Generally, mulches reduce competition from weeds, maintain soil temperature, and reduce evaporation from soil [2]. They protect the soil from wind-, water-, and traffic-induced erosion. Further, fugitive dust from soil is suppressed by mulching [3]. Mulches also improve soil properties by improving moisture retention capacity, releasing different nutrients, and enhancing biological activities [4]. Therefore, with the improved soil properties, plants grow better [5, 6].

Mulches are broadly classified into three main groups: organic, inorganic, and living mulches. Organic mulches are derived from organic substances, such as agricultural wastes (straw and rice husks), waste from wood industries (sawdust and barks), and green wastes (leaves and wood chips) [1]. Inorganic mulches include polyethylene film, gravel, bricks, and cobblestones. Living mulches include clover, Manila grass, ryegrass, dwarf lily turf, and other types of grasses [7]. Each type of mulch has a particular set of characteristics. The choice of mulch depends on the type of soil, climate, and nutritional requirements of plants [8]. In China, gravel (cobblestone) is the inorganic mulch widely used in landscapes [9].It reduces water evaporation, moderates soil temperature, and suppresses weeds [10, 11]. Further, barks are used as the major organic mulch in landscapes. The barks not only maintain soil moisture, reduce soil temperature fluctuations, release nutrients into the soil, but also help enrich landscape types [12, 13]. However, one of the major concerns in using organic mulch is its potential to reduce the mineral nitrogen content in the soil [14]. Various studies have reported that decaying organisms under organic mulches require immobilized mineral nitrogen [15]. The living mulches can enhance soil quality and conserve soil moisture content in landscapes. However, a few studies have demonstrated that the living mulches also compete with the plants for nutrients and water, thus limiting plant growth [16].

Mulches have been widely used in gardens and green belts along the roadside in developed countries [17–19]. They can improve plant growth in landscapes by enhancing soil quality, such as conserving soil moisture and increasing soil nutrients [20]. In China, mulches are used to cover bare soil only at a few places in landscapes, where most of the soil is uncovered, especially in tree pits [21].

The tree-pit soil is an important source of nutrients for the growth of plants [22]. However, the soil in urban tree pits in China is associated with many problems, such as a low degree of porosity and high soil pH and density, which limit tree growth to some extent [23]. In Beijing, the capital of China, the pH of urban soils might be elevated due to limestone inadvertently being introduced through discarded construction material and rubbles [24]. And particulate matter 2.5 (PM_2.5_) has become one of the serious environmental problems in Beijing. The soil dust originating from the bare soil—of the tree pits and edge of flower beds—is one of the sources of PM_2.5_ [25]. Therefore, covering the bare soil in tree pits with mulch not only improves soil properties but also inhibits the generation of soil dust from tree pits. Understanding the effect of various mulches on pit soil properties and tree growth is vital for applying appropriately. Few studies have evaluated the effect of various mulches on the soil quality and plant growth in urban tree pits, especially in China. The aim of the present study was to determine the effects of three types of mulches (organic, inorganic, and living) on the physical and chemical properties of soil, to identify the effects on the growth of street tree (*Sophora japonica*), and to provide preliminary suggestion for choosing suitable mulches based on our research.

## Material and methods

### Site description

The present study was conducted at Zhixin Road, Haidian District, Beijing, China (39°59′40″N, 116°22′18″E; altitude 43.5 m). The region has a typical temperate and monsoon climate with average annual temperature and rainfall of 12.5°C and 628.9 mm, respectively. The tree species in the tree pits along the roadside included *S*. *japonica* L., *Populus tomentosa* Carr., *Platycladus orientalis* L., and *Ginkgo biloba* L.

The tree pits with *S*. *japonica* located north of the road were chosen for the present study. The age of the trees were 12–15 years, with average height and trunk diameter of 12.2 m and 26.7 cm, respectively. The inter-tree spacing was 4 m and area of each tree pit was 1.44 m^2^ (1.2 m × 1.2 m). The soil of the tree pits was classified as brown soil (19.5% clay, 65.1% silt, and 15.4% sand), which was cleared manually before the study. The physical and chemical properties of the soil (0–10 cm, 10–20 cm, and 20–40 cm depth) in the tree pits were determined before mulching (Tables 1 and 2).

**Table 1:** Physical properties of the soil at different depths in the urban tree pits before the experiment.

Depth	Bulk density (g.cm^-3^)	Total porosity (%)	Macroporosity (%)	Microporosity (%)
0–10 cm	1.45±0.03a	45.15±2.13a	36.84±2.69a	8.31±1.80a
10–20 cm	1.50±0.05a	42.08±1.55a	35.09±0.79a	6.99±0.79a
20–40 cm	1.52±0.06a	41.54±1.91a	34.12±0.62a	7.42±0.62a

Values with different letters in the same column indicate significant differences between treatments (*p*<0.05, *n* = 3). Data are means ± standard deviation.

**Table 2:** Chemical properties of the soil at different depths in the urban tree pits before the experiment.

Depth	pH	Organic matter (g.kg^-1^)	Total N (g.kg^-1^)	Mineral N (mg.kg^-1^)	Available P (mg.kg^-1^)	Available K (mg.kg^-1^)
0–10 cm	8.32±0.31a	15.70±2.48a	1.15±0.05a	8.63±0.80ab	14.32±0.97a	89.15±3.60a
10–20 cm	8.28±0.41a	11.02±1.04a	1.02±0.11b	11.5±2.24a	13.79±0.99ab	88.09±6.56a
20–40 cm	8.66±0.42a	12.98±1.74a	0.94±0.10b	7.35±0.78b	12.76±1.74b	92.70±3.85a

Values with different letters in the same column indicate significant differences between treatments (*p*<0.05, *n* = 3). Data are means ± standard deviation.

### Experimental design

Six treatments were established for the present study: (1) unmulched bare soil (CK); (2) cobblestones (CB) (inorganic mulch) of thickness < 2 cm and diameter 1–3 cm; (3) water permeable bricks (WPB) (inorganic mulch)of size 15 cm × 15 cm × 6 cm; (4) pine barks (PB) (organic mulch), wood processing residues of *Pinus tabuliformis* Carr., of thickness < 1 cm and length < 6 cm (PB); (5) green waste compost (GWC) (organic mulch) from green waste. Green waste was obtained from municipal curbside collection, cut into pieces (0.5–1 cm particle size), and then subjected to fermentation. The moisture content of the raw material was adjusted to 60% (w/w) and carbon to nitrogen ratio was adjusted between 25 and 30 for optimal microbial activity during fermentation [26]; (6) turf grass (TG) (living mulch); *Ophiopogon japonicus var*.*nana*,a herb, has been widely used in landscaping in Northern China. It was chosen to be planted as turf grass in the present study, the height of the aerial part was 5–6 cm.

Each tree pit was laid with different mulch (up to 6 cm depth). A circle of diameter 6–10 cm larger than the trunk diameter was created around the base of the trunk without any mulch. This was done to avoid the limiting effect of mulch on tree growth. The turf grass *O*. *japonicus* was transplanted at a spacing of 10 cm. The experiment was a randomized complete block design with three replicates.

Studies have suggested that soil properties and tree growth could be determined to compare different mulching effect under the same conditions (in the botanic garden or at the institute, other environmental factors had no differences) after one-year’s study period [14, 27]. Considering the slow growth of the trees in the tree pits, two-years’ study period was used during this experiment. The study commenced on July 5, 2014 and finished at June 26, 2016. After laying the mulches, the tree pits were watered adequately. During the study period, anthropogenic activities, daily landscape maintenance by the government authorities (such as watering the trees every 3–7 days), and application of fertilizer were avoided.

### Soil sampling and analysis

The soil moisture content was measured at monthly interval during 2015. Three random soil samples at depths 0–10, 10–20, and 20–40 cm, respectively, were collected. The individual soil samples were then placed in sterile plastic sealing bags and transported to the laboratory, where they were oven-dried at 105°C for 8 h and used for further analysis.

Two-years after mulching, the soil was sampled from the tree pits on June 26, 2016. Three soil samples per tree pit at depths 0–10, 10–20, and 20–40, respectively, were collected in 100 cm^3^ volumetric containers and used to evaluate the physical properties of soil. Further, six random soil samples at depths 0–10, 10–20, and 20–40 cm were collected to evaluate the chemical properties of soil. All the soil samples were placed in sterile plastic sealing bags and transported to the laboratory in car refrigerators.

The soil sample used to evaluate the chemical properties was divided into three subsamples. The first subsample was stored at 4°C to analyze the moisture content. The second subsample was air-dried in a soil drying room and ground. The sample was then passed through a 2-mm sieve to remove pebbles, construction wastes, fine roots, and other foreign materials before the analyses. The third subsample was obtained by passing a portion of the second subsample through a 0.149-mm sieve.

The bulk density of the sampled soil was measured as the mass of oven-dried soil. The total porosity was assessed by measuring soil saturation (total volume of water-filled soil pores); microporosity was assessed using tension table and water column of 6 × 10^−3^ MPa; macroporosity was calculated as the difference between the total porosity and microporosity. All evaluations were performed according to the methodologies described by Embrapa (1997) [28].The second subsample of soil was used to determine the mineral nitrogen (N) content and pH. The mineral N was determined by alkali-hydrolytic diffusion method [29]. The soil pH was determined using a pH meter at a soil to water ratio of 2:5. The third subsample was used to estimate organic matter, available phosphorus (P), available potassium (K), and total N. The organic matter was measured by sulfuric acid-potassium dichromate wet oxidation, followed by titration with ferrous sulfate according to the procedure of Walkley-Black [30]. The available P in the soil was measured by the Olsen method. The available K in the soil was determined using a flame photometer after ammonium acetate extraction.

### Tree growth

The trunk diameter and tree height were determined once a year. The first measurement was recorded on June 20, 2015, with subsequent measurement on the day of soil sampling. The height of the tree was measured from the soil surface to the highest point of the tree crown using a Blume-Leiss altimeter. The diameter of the tree trunk was measured 15 cm above the soil surface using a tree caliper.

### Statistical analysis

All data were analyzed using the SPSS software package (version 20.0) (IBM Corporation, Armonk, New York). The data were subjected to one-way of variance (ANOVA) and means were separated by the least significant difference test at P < 0.05.

## Results and discussion

### Effect of different mulches on the moisture content of soil in the tree pits

**Fig 1:**
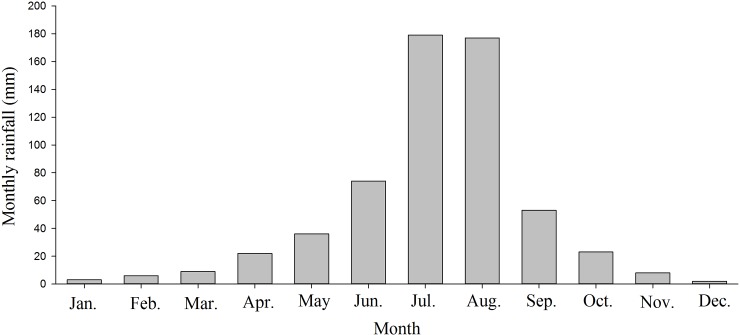
Monthly rainfall in Beijing, 2015.

The moisture content of the soil treated with different mulches was measured at depths 0–10, 10–20, and 20–40 cm from January to December 2015. The results indicated that at different depths, different mulches exhibited different effects on the soil moisture content. Further, during most months, the mulches significantly affected the moisture content of the soil at all the depths analyzed. Fig 1 represents the rainfall in Beijing in 2015. The study site also received similar amount of rain, with a relatively high rainfall in summer (July and August). Consequently, all the treatments exhibited relatively high moisture content in July and August.

**Fig 2:**
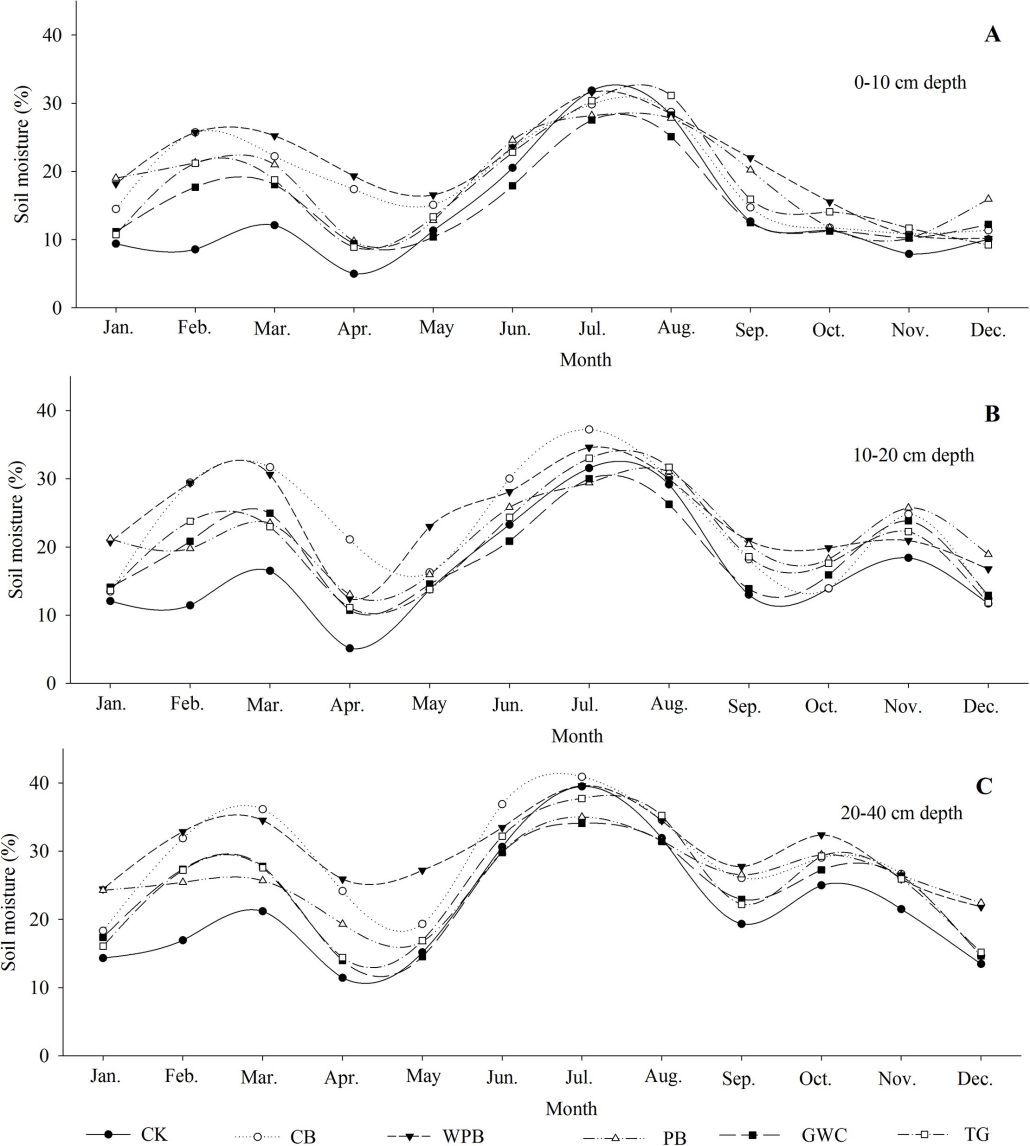
Monthly soil moisture content at three different depths in the urban tree pits treated with bare soil (CK), cobblestones (CB), water permeable bricks (WPB), pine barks (PB), green waste compost (GWC), and turf grass (TG) in 2015.

At 0–10 cm, during most months, the mulches increased the soil moisture content, except in July and August (Fig 2). Further, the effect of inorganic mulches (CB, WPB) on the soil moisture content was stronger than that of the organic mulches (PB, GWC) and living mulch (TG). In July and August, CB and WPB increased the soil moisture content. This might be associated with decreased rate of evaporation from the soil due to CB and WPB. However, the effect of other mulch types (PB, GWC and TG) on soil moisture content was lesser than that of CK. This might be due to the following reasons: (1) the organic mulches (PB, GWC) perhaps increased the rate of evaporation, and they absorbed water from the soil at tropical temperatures and (2) the TG and plants competed for water when the transpiration rate increased in plants due to hot weather. Similar results were observed at depths 10–20 and 20–40 cm, however the variations were significantly high.

Earlier studies have suggested that mulches, including plastic, gravel, barks, wood chips, and grass can retain the moisture content of soil by reducing the rate of evaporation [9, 31–33]. Several studies have revealed that living mulches can increase the moisture content of soil [27, 34], but because of the increasing rate of transpiration in plants, the opposite results were observed in the present study for living mulches in July and August.

### Effect of different mulches on the physical properties of soil in the tree pits

There was no significant difference in the bulk density of the soil treated with different mulches when compared with that of CK, except for PB, GWC, and TG treated soil at10–20 cm (Table 3). The treatments with PB, GWC, and TG significantly affected the bulk density at 10–20 cm when compared with that of CK, with GWC exhibiting a relatively strong effect. Some studies have shown that mulches can improve the bulk density of soil, but not significantly [27, 35]. At all the depths analyzed, WPB treated soil exhibited relatively low total porosity, macroporosity, and microporosity at the depths 0–10 and 10–20 cm. Relatively high total porosity, macroporosity, and microporosity were observed in GWC treated soil at 0–20 cm. Furthermore, at depths 0–10 and 10–20 cm, TG treated soil exhibited relatively high total porosity and macroporosity. These results might be due to many reasons: (1) when it rains, organic mulches (PB, GWC) might release organic matter creating a favorable condition for microorganism. Therefore, the porosity and bulk density of the soil at lower depths is improved [36]; (2)the root activity of TG—a living mulch—might have played an important role in improving the physical properties of the soil [27]; and (3) the use of high-density WPB as a mulch to cover bare soil can make the soil compact.

**Table 3:** Soil physical properties at three different depths in the urban tree pits treated with bare soil (CK), cobblestones (CB), water permeable bricks (WPB), pine barks (PB), green waste compost (GWC) and turf grass (TG) two-years after mulching.

Depth	Treatment	Bulk density (g.cm^-3^)	Total porosity (%)	Macroporosity (%)	Microporosity (%)
0–10 cm	CK	1.47±0.13ab	44.28±1.72b	36.47±1.54bc	7.81±0.38bc
	CB	1.51±0.03a	45.13±1.16b	35.97±1.18bc	9.16±0.30ab
	WPB	1.55±0.06a	40.64±1.29c	33.46±0.70c	7.18±0.65c
	PB	1.39±0.03ab	47.21±1.25ab	37.84±2.37ab	9.37±1.41ab
	GWC	1.34±0.04a	49.96±1.22a	39.67±1.63ab	10.29±0.45a
	TG	1.39±0.07ab	48.31±1.81a	40.96±2.26a	7.35±0.45c
10–20 cm	CK	1.52±0.05a	43.64±1.10bc	35.41±1.14b	8.23±0.34a
	CB	1.48±0.03ab	43.71±1.09bc	36.77±1.52ab	6.94±0.70a
	WPB	1.55±0.06a	41.27±1.76c	35.09±1.04b	6.18±1.64a
	PB	1.42±0.03bc	44.54±1.47bc	37.69±2.22ab	6.85±2.51a
	GWC	1.36±0.07c	48.72±1.87a	40.06±1.13a	8.66±0.78a
	TG	1.42±0.05bc	46.80±2.39ab	37.86±2.45ab	8.94±0.45a
20–40 cm	CK	1.50±0.09a	41.92±0.83a	35.02±1.02a	6.90±0.35a
	CB	1.52±0.03a	42.98±1.14a	35.84±1.83a	7.14±0.72a
	WPB	1.51±0.04a	41.76±1.87a	35.25±1.39a	6.51±0.85a
	PB	1.54±0.07a	42.76±1.15a	35.24±1.13a	7.52±0.60a
	GWC	1.55±0.07a	41.69±1.51a	35.18±1.24a	6.51±0.48a
	TG	1.53±0.07a	43.40±1.93a	35.98±1.50a	7.42±0.68a

Values with different letters in the same column indicate significant differences between treatments (*p*<0.05, *n* = 3). Data are means ± standard deviation.

### Effect of different mulches on the chemical properties of soil in the tree pits

**Fig 3:**
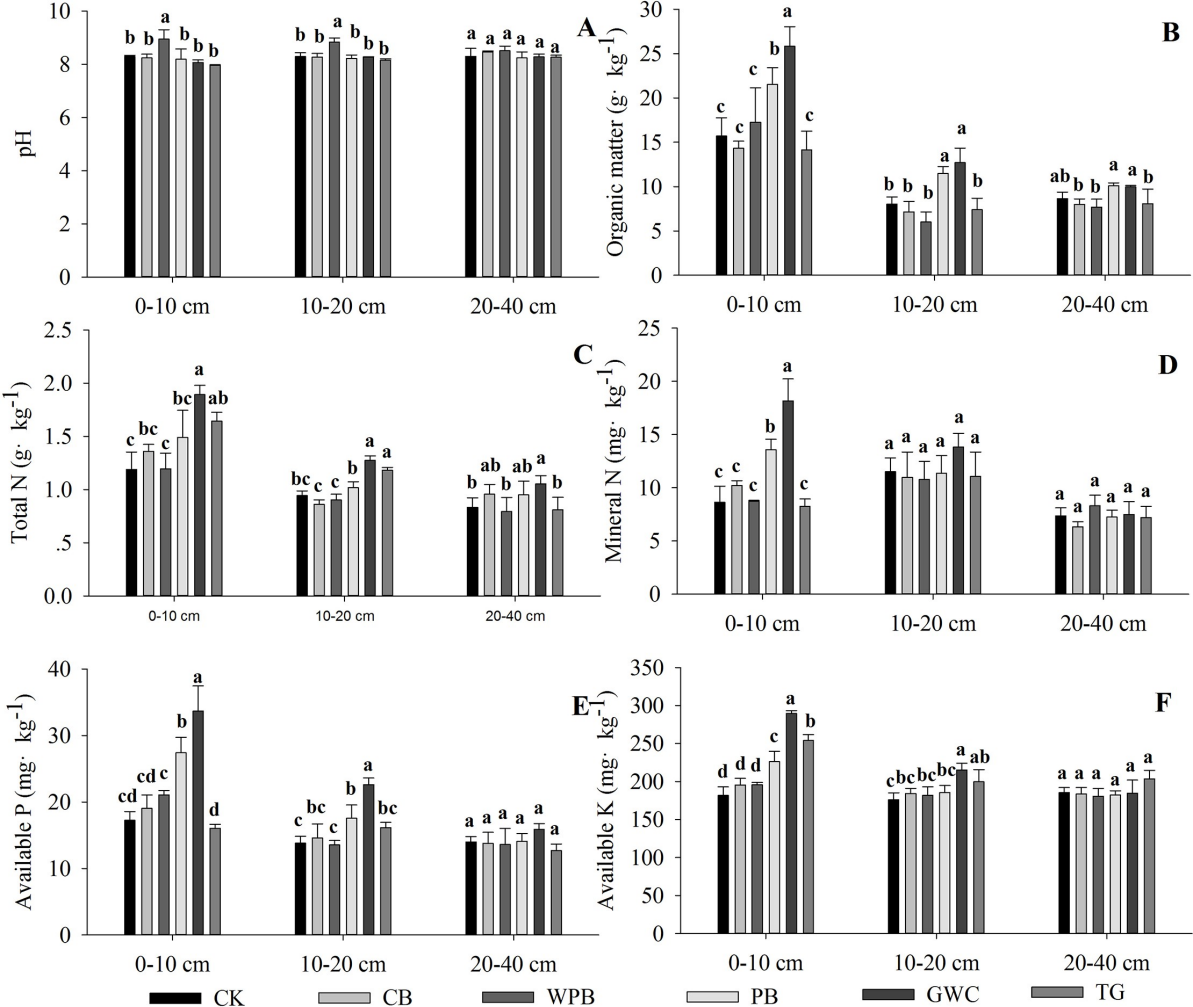
Soil chemical properties at three different depths in the urban tree pits treated with bare soil (CK), cobblestones (CB), water permeable bricks (WPB), pine barks (PB), green waste compost (GWC), and turf grass (TG) two years after mulching. Different letters above the error bars indicate significant differences between treatments (*p*<0.05, *n* = 3). The error bars indicate standard deviation.

The chemical properties of the soil two-years after mulching have been represented in Fig 3. The pH of the soil (Fig 3A) treated with WPB was significantly higher than that of CK. The pH of soil at depths 0–10 and 10–20 cm were 7.8% and 6.6% higher than that of CK. The relatively high pH observed was probably due to leaching of basic cations from WPB that are made of concrete. There was no significant difference in the soil pH among other treatments. Billeaud et al. [37] reported that mulches, such as wood chips, pine barks, and cypress, can significantly decrease the pH of fine sandy loam soil two-years after mulching. Pickering et al. [36] observed that mulching with cocoa shells, wood chips, garden compost, and horse manure can significantly increase the pH of sandy loam soil after one year. Qian et al. [7] reported that there was no significant difference in the soil pH between no mulch and living mulch treatments in the sandy loam soil of an apple orchard three-years after the treatment. The results of the present study were synonymous with these reports.

The organic matter in GWC and PB treated soils was significantly high at the depths 0–10 and 10–20 cm. Further, the organic matter in GWC treated soil was significantly higher than that of PB treated soil. Earlier studies have shown that organic mulches increase the soil organic matter by increasing biological activity in the soil [38, 39]. Similar results were observed in the present study. However, TG did not significantly affect the soil organic matter in the present study. This result contradicted earlier studies, which reported that grass significantly increased soil organic matter due to increased photosynthesis [27, 40].

The total N content of the soil treated with GWC and TG was significantly higher at the depths 0–10 and 10–20 cm when compared with that of CK. In GWC treated soil, significant increase of 37.1% and 26.1% were recorded at the depths of 0–10 and 10–20 cm, respectively. Further, PB and CB treatments also increased the total N content of the soil at 0–10 cm, however, the increase was not significant. The results revealed that the soil treated with organic mulches—GWC and PB—exhibited higher mineral N content when compared with that of CK, with GWC treated soil exhibiting a relatively high mineral N content. There was no significant difference among the treatments at the depths 10–20 and 20–40 cm.

The available P content was significantly affected only by the organic mulches—GWC and PB—at 0–10 cm. Further, GWC treatment showed a significant increase in the available P content at 10–20 cm. Although not significant, PB treatment also increased the available P content at 10–20 cm. Furthermore, GWC and PB treatment also significantly increased the available K content at 0–10 cm. Treatment with TG significantly increased the available K content at the depths 0–10 and 10–20 cm.

It was evident from the results that the differences in the mineral N, available P, and available K contents between treatments were similar to that of the organic matter. The favorable change in the available nutrients due to mulching with organic materials can be attributed to the increased biological activity in the soil. Thus, resulting in the mineralization of organic matter leading to increased nutrient content [41]. The treatment with GWC significantly increased not only the soil nutrients, but also total N. This might be because GWC was rich in nutrients. Therefore, the layer between GWC and soil supported a large number soil microbe population, which increased the enzyme activity in the region.

In the present study, the treatment with TG increased the total N content of the soil at lower depths two-years after mulching. This might be due to the activity of TG roots providing suitable condition for the growth of microorganisms that release N via organic matter decomposition [7].

### Effect of different mulches on the trunk diameter and height of the tree

**Fig 4:**
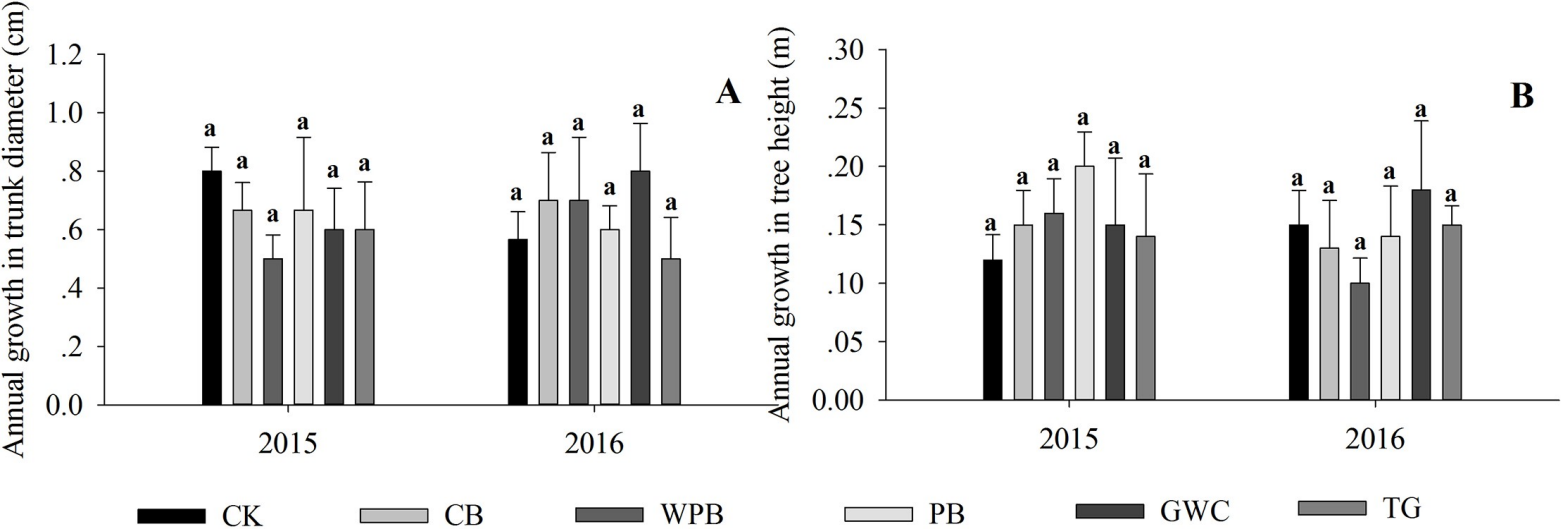
Annual increase in trunk diameter and tree height of trees in tree pits treated with bare soil (CK), cobblestones (CB), water permeable bricks (WPB), pine barks (PB), green waste compost (GWC) and turf grass (TG). Different letters above the error bars indicate significant differences between treatments (*p*<0.05, *n* = 3). The error bars indicate standard deviation.

The ANOVA revealed that the trunk diameter and height of *S*. *japonica* was not affected by different mulches. None of the treatments increased the trunk diameter and tree height significantly (Fig 4). Several studies have reported that mulching had no effect on tree growth. Ferrini et al. [42] observed that pine bark as mulch did not significantly affect the trunk diameter and height of ornamental trees. Iles et al. [43] demonstrated that organic mulches had no effect on the trunk diameter and height of red maple trees two-years after mulching. However, studies have also reported that mulching, especially with organic mulches, can improve growth and yield of plants [5, 44, 45]. The results observed in the present study might be attributed to the soil layer, where the tree roots were growing, not being affected by mulching. This can be validated by the physical and chemical properties of the soil, which did not differ significantly among the treatments at 20–40 cm, except for the soil moisture content.

Furthermore, factors, such as high average age of the trees, low inter-tree spacing distance, and lack of fertilizer, might have limited tree growth to some extent in urban tree pits. Therefore, further studies are necessary in this direction.

## Conclusions

The inorganic mulches (CB and WPB), organic mulches (GWC and PB) and living mulch (TG) exhibited positive effect by elevating the soil moisture content. However, the moisture content of soil applying GWC, PB, and TG decreased in July and August when compared with that of CK. PB, GWC, and TG improved the soil bulk density, total porosity, macroporosity, and microporosity of the soil to some extent at lower depths, whilst WPB worsened those factors. In addition, by comparing with other treatments, GWC significantly increased the level of all the nutrients. There was no significant difference in the soil properties among the treatments at 20–40 cm. Furthermore, the trunk diameter and tree height of S. *japonica* were not affected by the mulches. Organic mulches, especially GWC, seem to be better than other mulches analyzed to cover bare soil in the urban tree pits.
